# Improving uptake of lung cancer screening: an observational study on the impact of timed appointments and reminders

**DOI:** 10.1136/thorax-2024-222433

**Published:** 2025-02-13

**Authors:** Amyn Bhamani, Sindhu Bhaarrati Naidu, Tanya Patrick, Lavanya Anandan, Kiran Desai, Fanta Bojang, Priyam Verghese, Paul Robinson, Shivani Patel, Ricky Thakrar, Arjun Nair, Neal Navani, Sam M Janes

**Affiliations:** 1 Lungs for Living Research Centre, UCL Respiratory, University College London, London, UK; 2 University College London Hospitals NHS Foundation Trust, London, UK

**Keywords:** Lung Cancer

## Abstract

Lung cancer screening (LCS) reduces lung cancer-related mortality; however, uptake remains low compared with other cancer screening programmes. In this observational study, we report the impact of timed appointments and reminders on participation in our regional LCS programme.

Initial uptake of timed appointments was 53.0% (n=17 274/32 593), higher than previously reported in the UK, while initial uptake of open invitations was 29.8% (n=10 246/34 371). Among initial non-responders, 17.5% (n=4263/24 400) completed triage following a reminder. The increased participation following reminders only partially offset the significant difference in initial uptake between the two appointment types.

Timed appointments and reminders are strongly advocated to increase participation in national LCS programmes.

## Introduction

 Low-dose CT (LDCT) screening reduces lung cancer-related mortality; however, uptake of lung cancer screening (LCS) remains low. In the USA, uptake among eligible individuals in 2022 was 4.5%.^1^ While uptake in the UK is higher (20.4%–52.6%),^2 3^ it still lags behind other established national cancer screening programmes such as breast (64.6%)^4^ and bowel (68.9%).^5^ Participation is also notably lower among those most likely to benefit from screening,^3^ potentially exacerbating pre-existing healthcare inequalities.

Opt-out invitations using pre-allocated timed appointments and reminders provide a promising way forward to improve LCS participation, as demonstrated by their impact in other cancer screening programmes.^6^ Indeed, uptake of Lung Health Check (LHC) invitations using pre-allocated timed appointments in the Lung Screen Uptake Trial (LSUT) was 52.6%,^3^ considerably higher than other LCS studies to date, while in SUMMIT, proportionally more invitees responded after receipt of a reminder than the initial invitation.^7^ A direct comparison of uptake between opt-out (timed appointment) and opt-in (open invitation) strategies within a single programme has not been reported.

In this observational study, we examine the ‘real-world’ impact of timed appointments and reminders on participation in the North-Central London (NCL) Targeted Lung Health Check (TLHC) programme.

## Methods

Further methodological details are provided in online supplemental material. In brief, individuals aged 55–74 years with a history of smoking coded in their primary care record were initially invited for eligibility assessment using open invitations between 2 December 2022 and 6 July 2023. From 13 July 2023, the programme switched to timed appointments in order to improve participation following a lower than expected response to the open invitation strategy. This analysis includes all invited individuals split into two groups: those sent open appointments between 2 December 2022 and 6 July 2023, and those sent timed appointments between 13 July 2023 and 29 February 2024.

**Figure 1:**
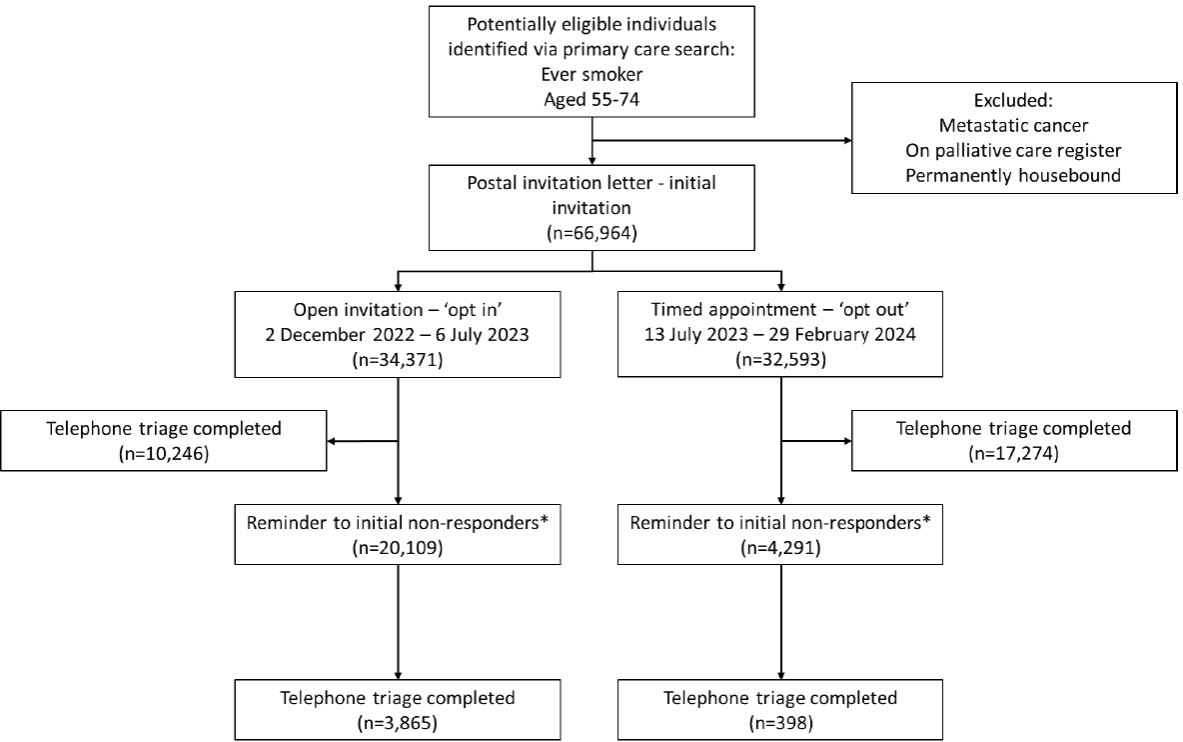
Flow diagram showing uptake of initial invitations and reminders.

Individuals sent open invitations were asked to contact the operations centre to complete an initial telephone triage. In the timed appointment strategy, individuals were given a pre-allocated appointment time during which the operations centre would call to complete triage. Non-responders in the open invitation strategy were defined as participants who did not contact the operations centre, and in the timed appointment strategy as those who did not answer their pre-allocated telephone call. Reminders were sent to initial non-responders in both strategies at least 4 (postal letter), 6 (text message) and 16 (postal letter) weeks later (figure 1).

During triage, individuals who identified as ‘never smokers’ and ‘never regular smokers’ were deemed ‘not eligible’ while those who had a thoracic CT in the past year had their screening deferred. These individuals did not proceed further with risk assessment.

This was an observational study. The primary outcome measures were the difference in response rate according to the primary explanatory variable of appointment type (open invitations vs timed appointments), and the additional benefit of sending reminders to initial non-responders in both groups. Secondary analyses explored the impact of these interventions when considering various participant characteristics associated with under-representation in screening.

## Results

### Response to initial invitations among all invitees

66 964 individuals were included in the analysis. Open invitations and timed appointments were used to invite 51.3% (n=34 371/66 964) and 48.7% (n=32 593/66 964) of individuals, respectively. 29.8% (n=10 246/34 371) of individuals sent an open invitation responded immediately (ie, without needing a reminder), compared with 53.0% (n=17 274/32 593) of those sent a timed appointment (p<0.0001) (table 1).

**Table 1:** Characteristics of individuals invited to a timed appointment compared with those invited with an open invitation

All invitees	Open invitation(n=34 371)	Timed appointment(n=32 593)	P value†
Age (median, IQR)*	63 (59–68)	63 (59–68)	**<0.0001**
Male sex*	19 549 (56.9%)	17 991 (55.2%)	**<0.0001**
IMD rank* (median, IQR)	12 523 (7221–20 017)	14 473 (8522–20 654)	**<0.0001**
IMD quintile*1 (most deprived)2345 (least deprived)Missing	7403 (21.5%)10 584 (30.8%)7436 (21.6%)5194 (15.1%)3698 (10.8%)56 (0.2%)	4741 (14.5%)9923 (30.4%)8449 (25.9%)6355 (19.5%)3024 (9.3%)101 (0.3%)	**<0.0001**
Response to initial invitationRespondedDid not respond	10 246 (29.8%)24 125 (70.2%)	17 274 (53.0%)15 319 (47.0%)	**<0.0001**
Eligibility at initial invitationEligibleNot eligibleScreening deferred due to recent CTDid not respond to initial invitation	3454 (10.0%)6518 (19.0%)274 (0.8%)24 125 (70.2%)	5176 (15.9%)11 390 (34.9%)708 (2.2%)15 319 (47.0%)	**<0.0001**

Non-parametric data are presented as medians (interquartile rangeIQR) and compared with Mann-Whitney U test, while categorical data are presented as proportions (in percentages) and compared with Chi-squaredχ2 test. Statistically significant values are in bold.

* From primary care record.

† P value adjusted for multiple testing according to Bonferroni method. Statistically significant values are in bold.

IMD, Index of Multiple Deprivation.

Following the initial invitation, significantly more individuals sent a timed appointment were eligible for screening compared with those sent an open invitation (15.9%, n=5176/32 593 vs 10.0%, n=3454/34 371). Multivariable logistic regression found that receiving a timed appointment was the factor most strongly associated with responding to an initial invitation (adjusted OR (aOR) 2.66, 95% CI 2.58 to 2.75, p<0.0001) (online supplemental table 1).

### Response to reminders among initial non-responders

17.5% (n=4263/24 400) of initial non-responders completed triage following a reminder; 19.2% (n=3865/20 109) of individuals who had been sent an initial open invitation completed triage following a reminder, compared with 9.3% (n=398/4291) who had received an initial timed appointment (figure 1).

Multivariable logistic regression analysis showed that individuals of black ethnicity (aOR 1.30, 95% CI 1.13 to 1.48, p<0.0001), those residing within the two most deprived national socioeconomic quintiles (quintile 1: aOR 1.85, 95% CI 1.60 to 2.16, p<0.0001; quintile 2: aOR 1.31, 95% CI 1.14 to 1.51, p<0.0001) and current smokers (aOR 1.28, 95% CI 1.18 to 1.39, p<0.0001) were more likely to respond after a reminder relative to individuals of white ethnicity, those residing in the least deprived quintile and former smokers respectively (table 2). There were no interactions between ethnic group and quintile in multivariable analysis (online supplemental table 2).

**Table 2:** Logistic regression analysis comparing characteristics of individuals who responded after a reminder with those who responded to the initial invitation (includes only current and former smokers with no recent thoracic CT imaging)

	Responded to initial invitation (n=20 072)	Responded after reminder (n=3194)	Univariable OR (OR, 95% CI)	P value	Adjusted OR* (95% CI)	P value
			Of responding after reminder compared with initial invitation			
Age†						
For every increasing year	–	–	0.98 (0.98 to 0.99)	**<0.0001**	0.99 (0.98 to 1.00)	**0.002**
Sex†						
Female	8961 (44.6%)	1373 (43.0%)	1.00 (ref)	–	1.00 (ref)	–
Male	11 110 (55.4%)	1821 (57.0%)	1.07 (0.99 to 1.15)	0.08	1.03 (0.96 to 1.12)	0.388
Missing	1 (<0.1%)	–	–	–	–	–
Smoking status‡						
Former smoker	15 078 (75.1%)	2176 (68.1%)	1.00 (ref)	–	1.00 (ref)	–
Current smoker	4994 (24.9%)	1018 (31.9%)	1.41 (1.30 to 1.53)	<0.0001	1.28 (1.18 to 1.39)	<0.0001
Ethnicity‡						
Asian	1898 (9.5%)	320 (10.0%)	1.14 (1.00 to 1.29)	**0.046**	1.08 (0.94 to 1.22)	0.266
Black	1339 (6.7%)	312 (9.8%)	1.57 (1.38 to 1.79)	**<0.0001**	1.30 (1.13 to 1.48)	**<0.0001**
Mixed	617 (3.1%)	104 (3.3%)	1.14 (0.91 to 1.40)	0.236	1.05 (0.85 to 1.30)	0.624
Other	544 (2.7%)	118 (3.7%)	1.46 (1.19 to 1.79)	**0.0003**	1.35 (1.10 to 1.65)	**0.004**
Prefer not to say	106 (0.5%)	32 (1.0%)	2.04 (1.35 to 2.99)	**0.0005**	1.91 (1.26 to 2.82)	**0.002**
White	15 568 (77.6%)	2308 (72.3%)	1.00 (ref)	–	1.00 (ref)	–
IMD Quintile†						
1 (most deprived)	2905 (14.5%)	748 (23.4%)	2.06 (1.78 to 2.39)	**<0.0001**	1.85 (1.60 to 2.16)	**<0.0001**
2	5732 (28.6%)	1008 (31.6%)	1.41 (1.23 to 1.62)	**<0.0001**	1.31 (1.14 to 1.51)	**<0.0001**
3	5191 (25.9%)	716 (22.4%)	1.10 (0.96 to 1.28)	0.183	1.06 (0.91 to 1.22)	0.474
4	3946 (19.7%)	432 (13.5%)	0.88 (0.75 to 1.03)	0.106	0.86 (0.73 to 1.01)	0.063
5 (least deprived)	2243 (11.2%)	280 (8.8%)	1.00 (ref)	–	1.00 (ref)	–
Missing	55 (0.3%)	10 (0.3%)	–	–	–	–

Statistically significant values are in bold.

* Adjusted for age at response, sex, smoking status, ethnicity and deprivation rank (categorised into quintiles). Participants responding after a reminder were those who did not respond to their initial invitation.

† From primary care record.

‡ Self-reported by participant at telephone triage.

IMD, Index of Multiple Deprivation.

Subgroup analysis of those sent an initial open invitation found that individuals who responded after a reminder differed in ethnicity, deprivation rank and smoking history compared with initial responders. Individuals initially sent a timed appointment who responded after a reminder differed in terms of ethnicity alone (online supplemental table 3).

## Discussion

In our clinical LCS service, initial uptake of timed appointments was 53%. In contrast, uptake of open invitations was significantly lower (30%) and the comparatively greater impact of reminders in this setting only partially offset the significant difference in initial uptake between the two strategies. Reminders did, however, improve uptake among groups generally under-represented in screening, including ethnic minorities.

These results are consistent with outcomes reported by UK-based LCS research studies to date. LSUT, the largest study to report data on uptake using timed appointments, reported overall uptake of 52.6%, while uptake of the first timed appointment (ie, prior to reminders) was 40.3%.^3^ Other studies, such as SUMMIT and pilots in Manchester and West London, used open invitations and uptake in these studies (20.4%–31%)^2 7 8^ was similar to our open invitation cohort. Finally, while overall uptake in the Yorkshire Lung Screening Trial (YLST) was higher (50.8%), only 28% of invitees responded to the initial open invitation, which is also in keeping with our findings.

Socioeconomic deprivation and current smoking status have consistently been identified as factors associated with lower participation in LCS.^9 10^ SUMMIT also demonstrated that individuals residing in areas of greater socioeconomic deprivation, current smokers and those of Asian, black and other ethnicities were more likely to respond to a reminder, rather than the initial invitation.^7^ Our analysis suggests that these findings are also applicable in a ‘non-research’ clinical service setting. One possible explanation is provided by the ‘Behaviour Change Wheel’ concept,^11^ which suggests three necessary conditions (capability, opportunity and motivation) to participate in LCS.^12^ Interventions such as reminders help address these conditions, for example, by providing further opportunities to participate.

The limitations of using primary care data to risk-stratify individuals for LCS have previously been demonstrated.^13^ In our cohort, 23% of individuals who completed telephone triage were never smokers or had never smoked regularly, despite a smoking code being present in their primary care record. These data highlight the need to improve the accuracy of primary care smoking records and, in turn, the efficiency of a national LCS programme. The NHS Health app and text messaging have previously been suggested as novel methods which may be of benefit in this regard.^14^

Limitations of this study include its observational nature and the sequential implementation of the two invitation strategies. Consequently, the higher uptake of timed appointments could reflect both differences in the populations and primary care practices being invited, and greater public awareness of LCS following a national press release announcing the roll-out of a national LCS programme in June 2023. The analysis could, therefore, have been strengthened by the availability of more detailed demographic data for non-responders. In addition, proportionally fewer non-responders in the timed appointment group had been sent reminders at the time of data analysis, possibly contributing to the observed lack of demographic difference between initial responders and those responding to a reminder in the timed appointment group. Nevertheless, despite these limitations, our findings provide ‘real-world’ insights which demonstrate the potential benefits of using timed appointments to improve LCS participation.

In summary, our study shows that timed appointments and reminders significantly increase participation in LCS. These interventions are strongly advocated to improve participation in national screening programmes.

## Supplementary material

10.1136/thorax-2024-222433 online supplemental file 1

10.1136/thorax-2024-222433 online supplemental file 2
