# Supplementary material for: Improving uptake of lung cancer screening: an observational study on the impact of timed appointments and reminders
Source: Thorax. 2025 Feb 13;80(5):e222433. doi: 10.1136/thorax-2024-222433 (PMC12015044; doi:10.1136/thorax-2024-222433)
Supplement: online supplemental file 1 [file thorax-80-5-s001.docx]

**Supplementary materials**

**Methods**

Primary care practices in North-Central London were invited sequentially. Practices who had not participated in SUMMIT, a large Lung Cancer Screening (LCS) study conducted in North- East and Central London between 2019 and 2023, were invited first to avoid overlap. The invitation letter and accompanying information leaflet were sent in English, but appropriately translated materials were available on request.

At telephone triage, individuals with a five-year lung cancer risk of ≥2.5% based on the Liverpool Lung Project (LLPv2.0) model or a six-year risk of ≥1.51% based on the Prostate, Lung, Colorectal and Ovarian (PLCOm2012) model [1], [2] were eligible to attend a face-to-face appointment to confirm eligibility prior to LDCT. Face-to-face appointments were held in the same locations for both open and timed appointments.

Data on age, sex and postcode were extracted from primary care records at the point of invitation. Postcode was used to derive national index of multiple deprivation (IMD) ranks, which were categorised into quintiles for analysis. Additional data, including self-reported smoking status and ethnicity, were collected during telephone triage and, therefore, were not available for non-responders. Data obtained from telephone triage including triage outcomes were recorded on a bespoke electronic database and extracted for analysis.

For the primary outcome of difference in uptake amongst all invitees, Mann-Whitney U and Chi-squared tests were used to analyse continuous and categorical data respectively. Post-hoc analysis was performed with Bonferroni correction to determine differences within multiple groups. In addition, univariable and multivariable logistic regression analysis was used to review factors associated with response amongst individuals. Co-variates were chosen a priori and included factors which may influence response to LCS [3], including age, sex and deprivation rank categorised into quintiles. To determine the impact of reminders, univariable and multivariable logistic regression analysis was used to compare characteristics of individuals responding immediately to an initial invitation with those who only responded after reminders. Additional co-variates for this analysis included smoking status and ethnicity as these data were available for responders. We also performed a post-hoc exploratory analysis to review the interaction between ethnic group and deprivation. Never smokers and individuals with recent thoracic CT were excluded from this analysis as they did not proceed with risk assessment following confirmation of smoking and imaging status. Complete case (listwise) analysis was used where there was missing data.

**Supplementary Table 1:**

Logistic regression analysis comparing characteristics of individuals who responded to an initial invitation with those who did not respond

|  | **Responded to initial invitation**  **(n=27,520)** | **Did not respond to initial invitation**  **(n=39,444)** | **Univariable odds ratio**  **(OR, 95% CI)** | **p-value** | **Adjusted odds ratio***  **(aOR, 95% CI)** | **p-value** |
| --- | --- | --- | --- | --- | --- | --- |
|  |  |  | **Of responding to an initial invitation** | | **Of responding to an initial invitation** | |
| **Age**† |  |  |  |  |  |  |
| For every increasing year |  |  | **1.03 (1.02-1.03)** | **<0.0001** | **1.03 (1.03-1.03)** | **<0.0001** |
| **Sex**† |  |  |  |  |  |  |
| Female  Male  Missing | 12,930 (47.0%)  14,589 (53.0%)  1 (<0.1%) | 16,493 (41.8%)  22,951 (58.2%)  - | 1.00 (ref)  **0.81 (0.79-0.84)**  **-** | -  **<0.0001**  **-** | 1.00 (ref)  **0.83 (0.80-0.86)**  **-** | -  **<0.0001**  **-** |
| **IMD Quintile**† |  |  |  |  |  |  |
| 1 (most deprived)  2  3  4  5 (least deprived)  Missing | 3,910 (14.2%)  7,827 (28.4%)  7,013 (25.5%)  5,485 (19.9%)  3,212 (11.7%)  73 (0.3%) | 8,234 (20.9%)  12,680 (32.1%)  8,872 (22.5%)  6,064 (15.4%)  3,510 (8.9%)  84 (0.2%) | **0.52 (0.49-0.55)**  **0.67 (0.64-0.71)**  **0.86 (0.82-0.91)**  0.99 (0.93-1.05)  1.00 (ref)  - | **<0.001**  **<0.001**  **<0.001**  0.705  -  - | **0.55 (0.52-0.59)**  **0.66 (0.62-0.70)**  **0.81 (0.76-0.86)**  **0.90 (0.85-0.96)**  1.00 (ref)  - | **<0.0001**  **<0.0001**  **<0.0001**  **0.002**  -  - |
| **Appointment type** |  |  |  |  |  |  |
| Timed  Open | 17,274 (62.8%)  10,246 (37.2%) | 15,319 (38.8%)  24,125 (61.2%) | **2.66 (2.57-2.74)**  1.00 (ref) | **<0.0001**  - | **2.66 (2.58-2.75)**  1.00 (ref) | **<0.0001**  - |

*Adjusted for age, sex, deprivation rank (categorised into quintiles) and appointment type

†From primary care record

**Supplementary Table 2:**

Logistic regression analysis comparing characteristics of individuals who responded after a reminder with those who responded to the initial invitation (includes only current and former smokers with no recent thoracic CT imaging), including an interaction term for ethnic group and quintile

|  | **Adjusted odds ratio***  **(aOR, 95% CI)** | **p-value** |
| --- | --- | --- |
|  | **Of responding after reminder compared to initial invitation** | |
| **Age** |  |  |
| For every increasing year | 0.99 (0.98-1.00) | 0.0003 |
| **Sex** |  |  |
| Female  Male | 1.00 (ref)  1.03 (0.96-1.12) | -  0.400 |
| **Smoking status** |  |  |
| Former smoker  Current smoker | 1.00 (ref)  **1.28 (1.18-1.39)** | -  **<0.0001** |
| **Ethnicity** |  |  |
| Asian  Black  Mixed  Other  Prefer not to say  White | 0.92 (0.58-1.40)  0.99 (0.29-2.53)  1.34 (0.61-2.61)  1.39 (0.63-2.72)  **3.59 (1.13-9.78)**  1.00 (ref) | 0.703  0.981  0.429  0.376  **0.017**  - |
| **IMD Quintile** |  |  |
| 1 (most deprived)  2  3  4  5 (least deprived)  Missing | **1.84 (1.55-2.18)**  **1.33 (1.13-1.56)**  1.04 (0.88-1.22)  0.85 (0.71-1.02)  1.00 (ref)  - | **<0.0001**  **0.001**  0.670  0.073  **-**  **-** |
| **Ethnicity x IMD Quintile** |  |  |
| Asian x 1  Asian x 2  Asian x 3  Asian x 4  Black x 1  Black x 2  Black x 3  Black x 4  Mixed x 1  Mixed x 2  Mixed x 3  Mixed x 4  Other x 1  Other x 2  Other x 3  Other x 4  Prefer not to say x 1  Prefer not to say x 2  Prefer not to say x 3  Prefer not to say x 4  White x 5 | 1.54 (0.94-2.58)  1.10 (0.68-1.84)  1.03 (0.62-1.75)  1.09 (0.62-1.92) 1.20 (0.46-4.15)  1.29 (0.49-4.45)  1.53 (0.57-5.36)  1.67 (0.58-6.09)  0.62 (0.27-1.54)  0.75 (0.35-1.78)  0.87 (0.39-2.12)  0.97 (0.39-2.51)  0.83 (0.37-1.99)  0.92 (0.42-2.18)  1.22 (0.55-2.90)  0.99 (0.39-2.61)  0.71 (0.20-2.77)  0.29 (0.07-1.16)  0.73 (0.19-3.00)  0.34 (0.06-1.64)  1.00 (ref) | 0.095  0.696  0.917  0.771 0.737  0.641  0.443  0.383  0.282  0.490  0.753  0.951  0.653  0.833  0.640  0.990  0.603  0.072  0.656  0.188  - |

*Adjusted for age at response, sex, smoking status, ethnicity, deprivation rank (categorised into quintiles) and an interaction term between ethnic group and deprivation quintile. Participants responding after a reminder were those who did not respond to their initial invitation.

**Supplementary Table 3:**

Multivariable logistic regression analyses comparing characteristics of individuals who responded after a reminder with those who responded to the initial open invitation and timed appointments
(includes only current and former smokers with no recent thoracic CT imaging)

|  | **Open invitation – multivariable analysis** | | | | **Timed appointment – multivariable analysis** | | | |
| --- | --- | --- | --- | --- | --- | --- | --- | --- |
|  | **Responded to initial invitation (n=7,903)** | **Responded after reminder**  **(n=2,890)** | **Adjusted odds ratio**  **(aOR, 95% CI)** | **p-value** | **Responded to initial invitation (n=12,169)** | **Responded after reminder (n=304)** | **Adjusted odds ratio**  **(aOR, 95% CI)** | **p-value** |
|  |  |  | **Of responding after reminder compared to initial invitation** | |  |  | **Of responding after reminder compared to initial invitation** | |
| **Age** |  |  |  |  |  |  |  |  |
| For every increasing year | - | - | **0.98 (0.97-0.99)** | **<0.0001** | - | - | 1.01 (0.99-1.03) | 0.527 |
| **Sex** |  |  |  |  |  |  |  |  |
| Female | 3,534 (44.7%) | 1,235 (42.7%) | 1.00 (Ref) | - | 5,427 (44.6%) | 138 (45.4%) | 1.00 (Ref) | - |
| Male | 4,368 (55.3%) | 1,655 (57.3%) | 1.04 (0.95-1.14) | 0.382 | 6,742 (55.4%) | 166 (54.6%) | 0.89 (0.71-1.13) | 0.351 |
| Missing | 1 (<0.1%) | - | - | - | - | - | - | - |
| **Smoking status** |  |  |  |  |  |  |  |  |
| Former smoker | 6,071 (76.8%) | 1,953 (67.6%) | 1.00 (Ref) | - | 9,007 (74.0%) | 223 (73.4%) | 1.00 (Ref) | - |
| Current smoker | 1,832 (23.2%) | 937 (32.4%) | **1.42 (1.29-1.57)** | **<0.0001** | 3,162 (26.0%) | 81 (26.6%) | 0.99 (0.76-1.29) | 0.938 |
| **Ethnicity** |  |  |  |  |  |  |  |  |
| Asian | 614 (7.7%) | 272 (9.4%) | **1.18 (1.01-1.38)** | **0.032** | 1,284 (10.6%) | 48 (15.8%) | **1.77 (1.28-2.46)** | **0.001** |
| Black | 584 (7.4%) | 288 (10.0%) | **1.17 (1.01-1.37)** | **0.041** | 755 (6.2%) | 24 (7.9%) | 1.51 (0.98-2.34) | 0.063 |
| Mixed | 290 (3.7%) | 90 (3.1%) | 0.83 (0.65-1.06) | 0.141 | 327 (2.7%) | 14 (4.6%) | **2.03 (1.16-3.53)** | **0.013** |
| Other | 199 (2.5%) | 102 (3.5%) | **1.35 (1.06-1.73)** | **0.016** | 345 (2.8%) | 16 (5.3%) | **2.20 (1.30-3.71)** | **0.003** |
| Prefer not to say | 48 (0.6%) | 30 (1.0%) | 1.58 (0.99-2.52) | 0.054 | 58 (0.5%) | 2 (0.6%) | 1.64 (0.40-6.78) | 0.493 |
| White | 6,168 (78.0%) | 2,108 (72.9%) | 1.00 (Ref) | - | 9,400 (77.2%) | 200 (65.8%) | 1.00 | - |
| **IMD quintile** |  |  |  |  |  |  |  |  |
| 1 (most deprived) | 1,360 (17.2%) | 709 (24.5%) | **1.89 (1.60-2.24)** | **<0.0001** | 1,545 (12.7%) | 39 (12.8%) | 0.97 (0.60-1.58) | 0.898 |
| 2 | 2,251 (28.5%) | 924 (32.0%) | **1.54 (1.31-1.81)** | **<0.0001** | 3,481 (28.6%) | 84 (27.6%) | 0.95 (0.62-1.45) | 0.808 |
| 3 | 1,890 (23.9%) | 614 (21.2%) | **1.28 (1.08-1.51)** | **0.004** | 3,301 (27.1%) | 102 (33.6%) | 1.23 (0.81-1.86) | 0.332 |
| 4 | 1,354 (17.1%) | 384 (13.3%) | 1.16 (0.97-1.39) | 0.109 | 2,592 (21.3%) | 48 (15.8%) | 0.75 (0.47-1.19) | 0.215 |
| 5 (least deprived) | 1,036 (13.1%) | 250 (8.7%) | 1.00 (Ref) | - | 1,207 (9.9%) | 30 (9.9%) | 1.00 | **-** |
| Missing | 12 (0.2%) | 9 (0.3%) | - | - | 43 (0.4%) | 1 (0.3%) | - | **-** |

*Adjusted for age at response, sex, smoking status, ethnicity and deprivation rank (categorised into quintiles). Participants responding after a reminder were those who did not respond to their initial invitation

**References:**

[1] M. C. Tammemägi *et al.*, “Selection Criteria for Lung-Cancer Screening,” *New England Journal of Medicine*, vol. 368, no. 8, pp. 728–736, Feb. 2013, doi: 10.1056/NEJMoa1211776.

[2] O. Y. Raji *et al.*, “Predictive Accuracy of the Liverpool Lung Project Risk Model for Stratifying Patients for Computed Tomography Screening for Lung Cancer,” *Ann Intern Med*, vol. 157, no. 4, p. 242, Aug. 2012, doi: 10.7326/0003-4819-157-4-201208210-00004.

[3] N. Ali *et al.*, “Barriers to uptake among high-risk individuals declining participation in lung cancer screening: a mixed methods analysis of the UK Lung Cancer Screening (UKLS) trial,” *BMJ Open*, vol. 5, no. 7, p. e008254, Jul. 2015, doi: 10.1136/bmjopen-2015-008254.
