# Supplementary material for: Improving uptake of lung cancer screening: an observational study on the impact of timed appointments and reminders
Source: Thorax. 2025 Feb 13;80(5):e222433. doi: 10.1136/thorax-2024-222433 (PMC12015044; doi:10.1136/thorax-2024-222433)
Supplement: online supplemental file 2 [file thorax-80-5-s002.pptx]

## Slide 1
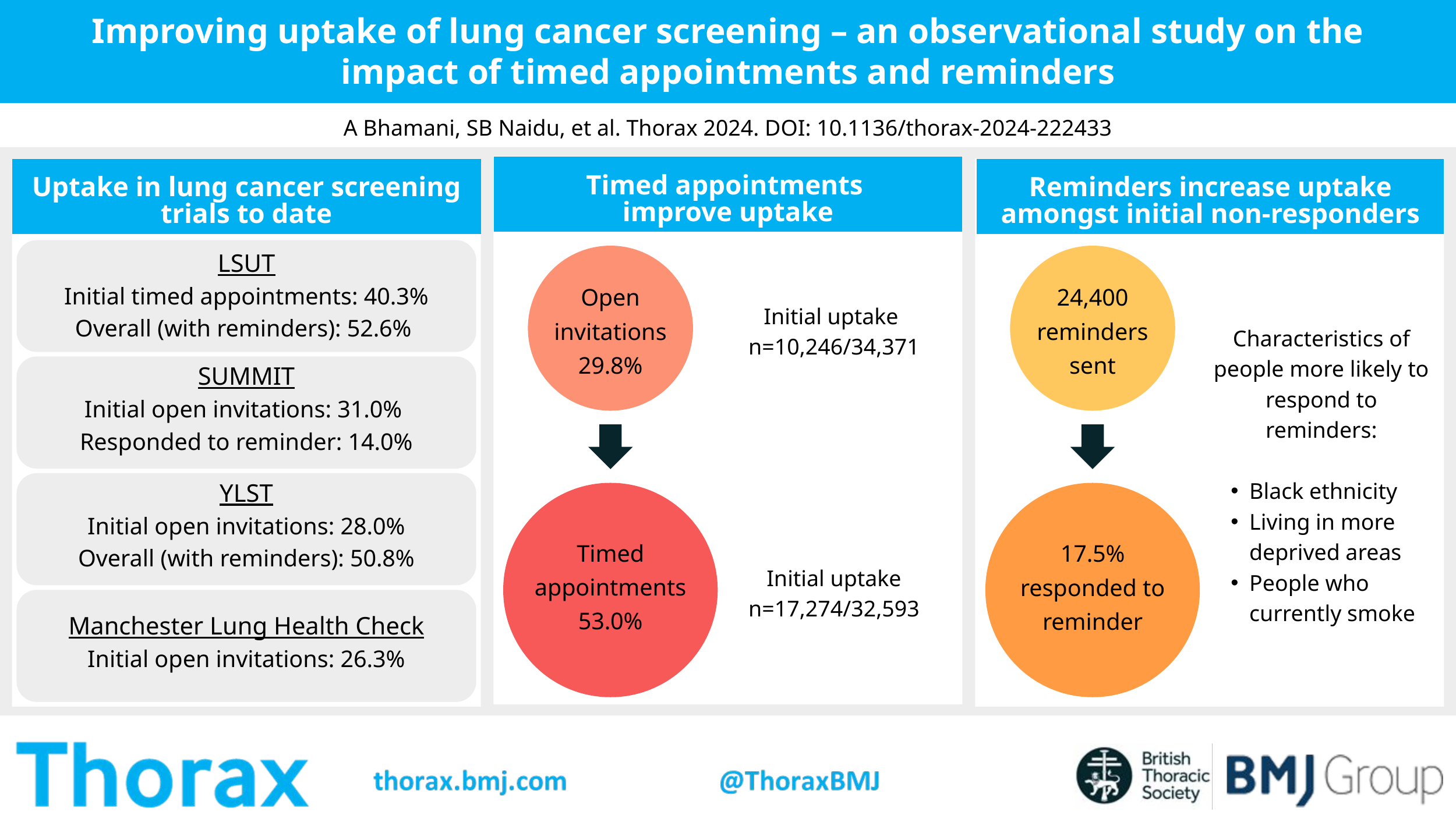

Improving uptake of lung cancer screening – an observational study on the impact of timed appointments and reminders
A Bhamani, SB Naidu, et al. Thorax 2024. DOI: 10.1136/thorax-2024-222433
Timed appointments
improve uptake
Uptake in lung cancer screening trials to date
Reminders increase uptake amongst initial non-responders
LSUT
Initial timed appointments: 40.3%
Overall (with reminders): 52.6%
Open invitations 29.8%
24,400 reminders sent
Initial uptake
n=10,246/34,371
Characteristics of people more likely to respond to reminders:
Black ethnicity
Living in more deprived areas
People who currently smoke
SUMMIT
Initial open invitations: 31.0%
Responded to reminder: 14.0%
YLST
Initial open invitations: 28.0%
Overall (with reminders): 50.8%
Timed appointments 53.0%
17.5% responded to reminder
Initial uptake n=17,274/32,593
Manchester Lung Health Check
Initial open invitations: 26.3%
